# A novel thermal compression device for perioperative warming: a randomized trial for feasibility and efficacy

**DOI:** 10.1186/s12871-017-0395-2

**Published:** 2017-08-11

**Authors:** Peter Luke Santa Maria, Chloe Santa Maria, Andreas Eisenried, Nathalia Velasquez, Brian Thomas Kannard, Abhinav Ramani, David Mark Kahn, Amanda Jane Wheeler, John Gerhard Brock-Utne

**Affiliations:** 1 Department of Otolaryngology, Head and Neck Surgery, Stanford University, 801 Welch Rd, Stanford, CA 94305 USA; 2 Department of Anesthesiology, Stanford University, Stanford, USA; 3 Department of Plastic Surgery, Stanford University, Stanford, USA; 4 Department of General Surgery, Stanford University, Stanford, USA; 5 Biodesign, Stanford University, Stanford, USA

**Keywords:** Perioperative normothermia, Thermal compression, Perioperative warming, Forced air warming

## Abstract

**Background:**

Inadvertent perioperative hypothermia (IPH) leads to surgical complications and increases length of stay. IPH rates are high with the current standard of care, forced air warming (FAW). Our hypothesis is that a prototype thermal compression device that heats the popliteal fossa and soles of the feet, with lower leg compression, increases perioperative temperatures and reduces IPH compared to the current standard of care.

**Methods:**

Thirty six female breast surgery patients, at a tertiary academic hospital, were randomized to the device or intraoperative FAW (stage I) with a further 18 patients randomized to the device with a single heating area only (stage II, popliteal fossa or sole of the feet). Stage I: 37 patients recruited (final 36). Stage II: 18 patients recruited (final 18). Inclusion criteria: general anesthesia with esophageal monitoring for over 30 min, legs available and able to fit the device and no contraindications to leg heating or compression. The intervention was*:* Stage I: Investigational prototype thermal compression device (full device group) or intraoperative FAW. Stage II: Device with only a single heating location. Primary outcomes were perioperative temperatures and incidence of IPH. Secondary outcomes were local skin temperature, general and thermal comfort scores and presence of perioperative complications, including blood loss.

**Results:**

Mean temperatures in the full device group were significantly higher than the FAW group in the pre-operative (36.7 vs 36.4 °C, *p* < 0.001), early intraoperative (36.3 vs 35.9 °C, *p* < 0.001), intraoperative (36.6 vs 36.2 °C, *p* < 0.001) and postoperative periods (36.8 vs 36.5 °C, *p* < 0.001). The incidence of IPH in the device group was also significantly lower (16.7% vs 72.0%, *p* = 0.001). Thermal comfort scores were significantly higher in the full device group and hypothermia associated wound complications were higher in the FAW group.

**Conclusions:**

The thermal compression device is feasible and has efficacy over the FAW. Further studies are recommended to investigate clinically significant outcomes.

**Trial registration:**

clinicaltrials.gov (NCT02155400)

**Electronic supplementary material:**

The online version of this article (doi:10.1186/s12871-017-0395-2) contains supplementary material, which is available to authorized users.

## Background

Inadvertent perioperative hypothermia (IPH) is defined as perioperative unintended core body temperature less than 36°C and occurs in up to 70% without preventative measures and in at least 20% with the best available care [1–3]. Risk factors include American Society for Anesthesiology (ASA) grade, combined regional and general anesthesia and intermediate or major surgery [2, 4]. IPH is associated with cardiac morbidity, wound infection, intraoperative blood loss and increased post-operative care unit (PACU) and hospital length of stay [1, 5, 6].

The standard of care for preventing IPH is intraoperative forced air warming (FAW). FAW has usability issues that affect compliance and as it relies on surface area coverage, is less effective in core surgery (involving the chest or abdomen) and can get in the way of the surgical site in core surgery [7, 8]. Even with full compliance, intraoperative FAW does not prevent IPH [9, 10]. This is because the critical period to prevent IPH is the preoperative phase [9]. Without preoperative warming, cold blood from the peripheries flows into the core on induction due to loss of peripheral vascular tone. There have been efforts to implement preoperative FAW with limited success [7, 11–19]. They are cumbersome, not easily transported with the patient and can easily and unknowingly be disconnected during transit to the operating room [7, 20–22].

There is a need for an effective perioperative warming device, placed in the preoperative period, which both warms and maintains clinical access during core surgery. Our hypothesis is that a prototype device providing heat to the popliteal fossa and soles of the feet, together with lower leg compression, is feasible and will have efficacy in maintain perioperative temperatures better than the current standard of care.

## Methods

The study and consent process was approved by Stanford’s Internal Review Board (Protocol 28,535). All patients were consented in person and signed the informed consent form. The trial was registered at clinicaltrials.gov (May 2014, Santa Maria, NCT02155400).

### Trial design

**Fig. 1:**
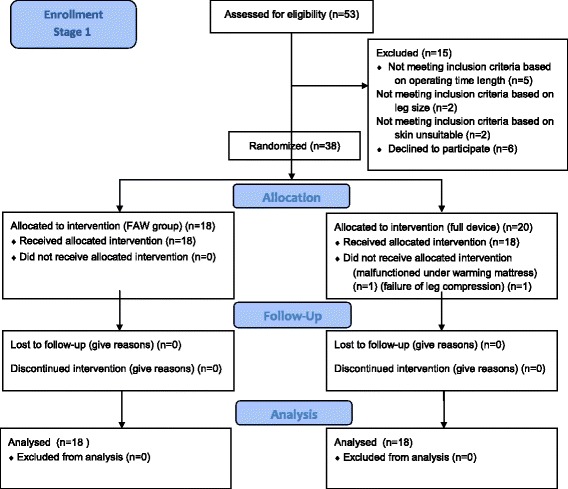
Shows the trial enrollment, allocation, follow-up and analysis according to the CONSORT standards. Groups are full device, forced air warming (FAW) group, popliteal group and sole group

The trial was designed according to CONSORT standards (see Fig. 1). Inclusion criteria included breast surgery under general anesthesia with an esophageal temperature probe, length of surgery for more than 30 min, legs fitting the sleeve dimensions of the device, two accessible legs and no contraindications to heating or compression of the lower legs. Recruitment occurred at Stanford University hospital from October 2014–March 2015. Randomization was done on a 1:1 ratio using a blocked randomization method and computerized number generator [23]. The elective breast surgery list was obtained weekly and the randomization was performed for that week. The block size was different each week and was dependent on the number of patients booked for surgery. The research assistant collecting the data was blinded to the randomization until the night before and the anesthesiologist and surgeon where blinded until they saw the patient in the preoperative bay. All people in the treating team were blinded to the randomization process. In the second stage of the study, using the same method, cases were assigned to either sole of the foot only or popliteal fossa only. Patients in all groups were operated in the supine position, received other standard of care measures including warmed blankets, warmed aesthetic gases, warmed intravenous fluids, and a thermal under warming mattress (Maxi-Therm Lite, 42 °C, Cincinnati Sub-zero, Cincinnati, USA) in the operating room. The operating room temperature was regulated between 21 and 23 °C. All patients received systemic antibiotic therapy on induction.

### Stage I – Full device group (18 patients)

**Fig. 2:**
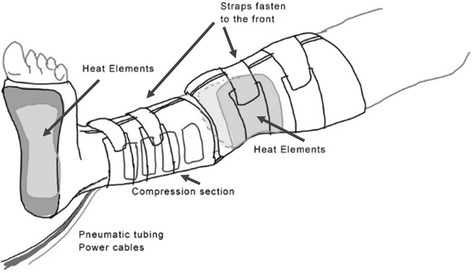
Artistic rendition of the prototype device. Figure 2 displays an artistic rendition of the prototype device identifying the areas of heating and compression within the lower leg sleeve. The heating elements are located at the popliteal fossa and sole of the foot strapped in close contact to the patient. Beneath the heating elements, in each location, are temperature feedback probes that feed back to the individual heating unit for that region. There is also a sequential pneumatic calf compression sleeve component fitted to the patient’s lower legs in between the two heating areas

For this group an investigational prototype thermal compression device was used. The device is placed on the patient in the preoperative bay during completion of the preoperative checklist, during which sequential compression devices are routinely placed on the patient, and is switched on for use for at least 30 min preoperative depending on the waiting time for transfer to the operating room. A more detailed technical description is included in Additional file 1. The device has 150 mm × 200 mm warming pads driven by resistive heating with a temperature probe held against the skin that feeds back to a controller unit to ensure the skin temperature is limited at 43 °C. The warming system is a closed loop control system where a temperature set point can be selected on the control unit and closed loop feedback and control occur between the temperature probe, control unit, and warming blanket. This allows the system to adjust the warming blanket temperature to ensure the delivery of appropriate thermal energy to the site for maximum warming while preventing any undesired complications due to the delivery of excess heat. Each warming site utilized a separate system with individual closed loop heating control to account for differences in warming blanket positioning, heat transfer, and other differences between the sites. The heating elements are fitted within pockets of sleeves that are then strapped in close contact to the patient in the popliteal fossa and sole of the foot. There is also a sequential pneumatic calf compression sleeve component fitted to the patient’s lower legs in between the two heating areas. Leads from the two heating areas and one compression unit feed via cables to a mobile controller unit that can be transported with the patient. Figure 2 displays an artistic rendition of the prototype device identifying the areas of heating and compression within the lower leg sleeve. In the operating room FAW (Bair Hugger, 3 M, St Paul USA) was placed on the body from the surgical preparation area site (upper abdomen) down but not turned on. One patient was excluded due to failure of the thermal under warming mattress at the beginning of surgery; another was excluded due to not having leg compression (discussed below).

### Stage I - FAW group (18 patients)

These patients had the current standard of care at Stanford Hospital with FAW (Bair Hugger Model 750, blanket Full Body, High setting (43 +/− 3 °C), 3 M, St Paul USA) placed on the body from the surgical preparation area site (upper abdomen) down and including the feet and activated as soon as possible once inside the operating room. The FAW blanket was removed after extubation and prior to transport of the patient to the PACU. FAW patients also had pneumatic sequential compression devices (Kendall SCD, Covidien, Mansfield, USA) placed as prophylaxis for deep venous thrombosis while in the operating room. No patients were excluded.

### Stage II - popliteal group or sole group

To understand the effect of individual heating areas, patients were randomized to groups with single heating areas only using the same above described process. Each group received the same interventions and standard of care as the device group in stage I but one group (9 patients) had only the popliteal fossae heated (popliteal group) while the other group (9 patients) was heated only with the sole of the feet (sole group). As the FAW group from stage I is the current standard of care at Stanford University Hospital, we intended to compare the results of those patients recruited in stage 2 with the results of the patients in the FAW group of stage I. Early feedback to the investigators from the attending anesthesiologists were that they were observing patients in these groups were needing to have the FAW activated often so we analyzed the results of this stage after our intended half way point (18 patients, 9 in each group). There was one (of nine) patient in the popliteal and two (of nine) patients in the sole groups that had their intraoperative temperature rescued, because of intraoperative hypothermia, using FAW. The incidence of perioperative hypothermia in the popliteal group (44%, *p* = 0.16) and sole group (55.6%, *p* = 0.39) and as it was not significantly different to FAW (72%) it was decided to no longer recruit into this stage.

### Outcomes

Our primary outcomes were perioperative temperatures and IPH incidence. Core temperature was estimated using a tympanic thermometer (Genius 2, Covidien. USA) during the whole perioperative period and measured, under anesthesia, using an esophageal temperature probe (placed 30-35 cm into the esophagus). Temperature at the skin was measured using a direct thermometer (Sure Temp Plus, Welch Allyn, USA). In the event that intraoperative hypothermia occurred in any of the device groups, it was up to the attending anesthesiologist, on a case by case basis, whether the FAW was turned on. Our secondary outcomes were local skin temperature, general and thermal comfort scores, intraoperative blood loss and presence of perioperative complications (including arrhythmia, post-operative seroma and wound infection). Thermal comfort was assessed using a visual analogue scale from −10 (uncomfortably cold) to 0 (comfortable) to 10 (uncomfortably warm). General comfort was assessed using a visual analogue scale from 0 (as uncomfortable as imaginably possible) to 10 (as comfortable as imaginably possible). Blood loss was recorded from the post-operative case note. Patients were also visited on the next post operative day prior to discharge. The anesthetic chart and medical notes where read retrospectively three months after the surgery for the presence of perioperative complications. The surgeons reviewing the patients postoperatively were blinded to which group the patient belonged to.

### Statistical analysis

Calculations were made with STATA version 13. A two-tailed paired T test compared means in temperature with post hoc Bonferroni corrections. Pearson’s Chi squared test for goodness of fit was used to compare incidence of hypothermia. The correlation of esophageal and tympanic temperature measurements was analyzed using least squares regression analysis with Pearson’s correlation coefficient. Relative risk was used to calculate the risk of postoperative wound complications in patients who had postoperative hypothermia. Sample sizes were determined using a predicted efficacy of each group calculated with a minimum α of 0.05 and β of 0.8 for the primary outcomes involving temperature measurement (perioperative temperatures during each phase of surgery and IPH incidence). We took the lower incidence of IPH in large cohorts using FAW to have been reported as 60% [1–3].. We made the assumption for an incidence of IPH in the full device arm to be 20%. Our calculation gave us a cohort of 18 patients in each treatment arm. Power was achieved in all reported outcomes except those mentioned.

## Results

Table 1 displays demographics and surgery details. Patients were female only. There was no difference in age, BMI, operating time, ASA grade, between full device and FAW groups. The correlation of esophageal and tympanic temperature measurement taken during the intra-operative period was *r* = 0.88 (Pearson’s correlation coefficient, *p* < 0.0001) and this supports the tympanic measurements as a surrogate for core temperatures in awake patients.

**Table 1:** Demographics

Demographics		Full device (*n* = 18)	FAW (*n* = 18)	Popliteal (*n* = 9)	Sole (*n* = 9)
Age in years		53.6 (4.3)	52.9 (2.8)	47.6 (5.3)	39.1 (3.7)
BMI		30.1 (2)	27 (1.1)	30.6 (2.2)	25.3 (2.3)
ASA class (median, IQR)		2 (1)	2 (1)	2 (1)	2 (1)
Premedication given preoperatively		12/18	12/18	9/9	8/9
Operation length (minutes)		163.1 (23)	152.1 (17.4)	198.6 (15.1)	140.9 (31.9)
Major diagnosis	Cancer	13	16	7	7
	Benign neoplasm	1	1	0	0
	Cosmetic	2	1	1	1
	Gender reassignment	2	0	1	1
Surgery type	Bilateral reconstruction/Insertion of breast implants	1	0	1	3
	Bilateral breast reduction	2	1	4	1
	Lumpectomy +/− axillary node dissection	3	4	1	0
	Unilateral mastectomy +/− axillary node dissection +/− tissue expanders	6	9	1	2
	Unilateral mastectomy with pedicled muscle flap	1	1	0	0
	Bilateral mastectomy +/− axillary node dissection +/− tissue expanders	5	3	2	3

Table 1 displays the demographic data for the cohort. There were no significant differences in the cohort between the full device and the forced air warming (FAW) groups. Patients were younger in the popliteal and sole groups however the cohort sizes are small. Standard deviation is displayed in parentheses Abbreviations are *BMI* (body mass index), *ASA* (American Society of Anesthesiologists)

### Peri-operative temperatures in treatment group

**Fig. 3:**
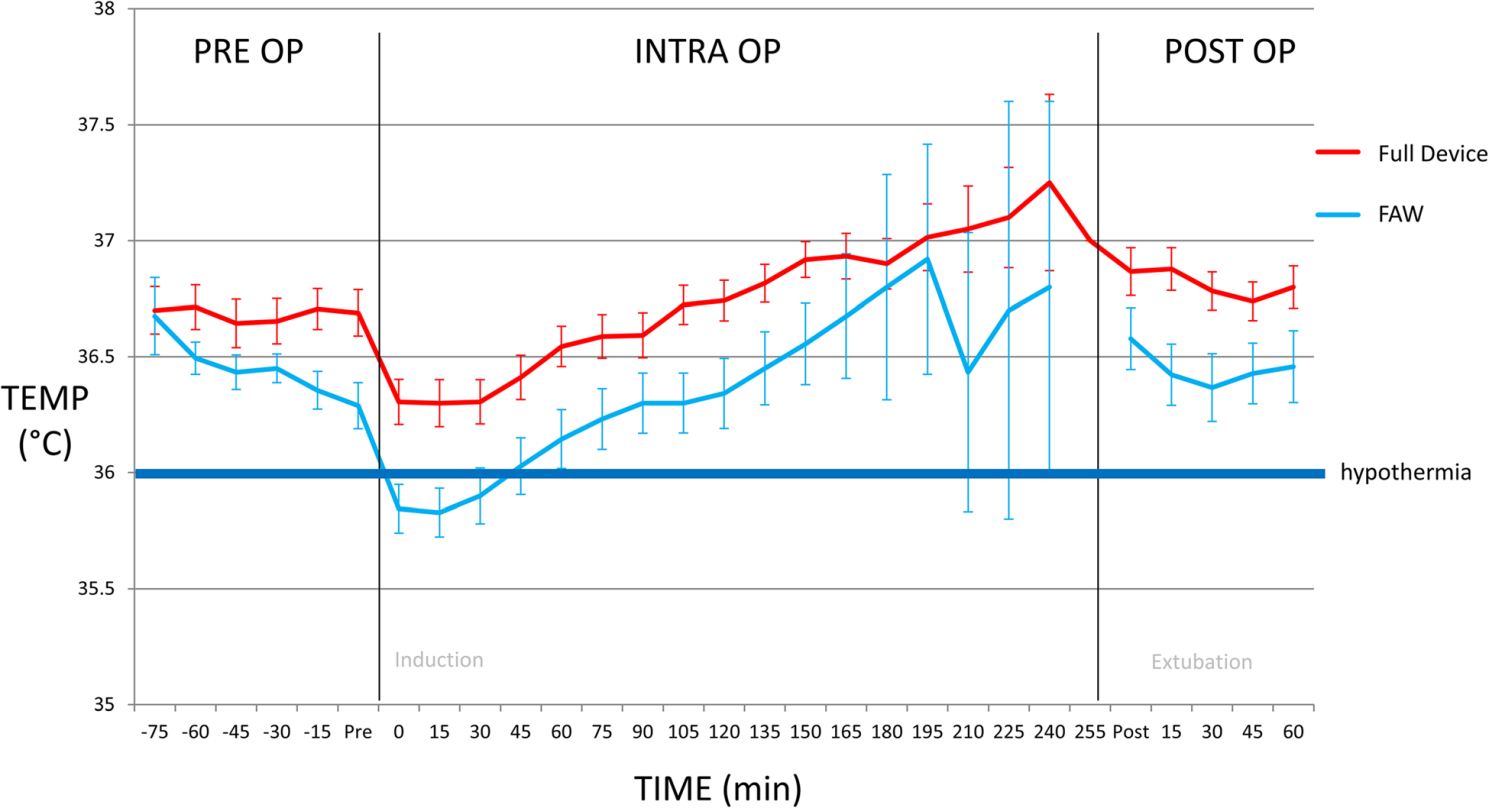
Perioperative Temperatures. Figure 3 shows the temperature benefit of the full device over the FAW. The mean patient temperatures (temp) with standard error are shown for the full device (*n* = 18) and FAW (*n* = 18) treatment groups. Each phase is shown including pre op (preoperative), intra op (intraoperative) and post op (postoperative). Patient in the full device group had higher mean temperatures in each phase of surgery compared to the FAW group where patient became hypothermic soon after induction

The perioperative temperatures are displayed in Fig. 3 and Table 2. Mean temperatures in the full device group were significantly higher than the FAW group in the pre-operative, intraoperative and postoperative periods. The incidence of perioperative hypothermia (core temperature below 36 °C) in the full device group was 16.7%. This was significantly lower than the incidence in the FAW group which was 72% (*p* = 0.001). There was one patient in the full device group that received the FAW shortly after induction of anesthesia at the bequest of the attending anesthesiologist. There was also a significant difference in the incidence of post-operative hypothermia with the device, having a lower incidence compared to the FAW group (0% vs 22.2%, *p* = 0.03).

**Table 2:** Temperature results

Period	Treatment group	Mean temp °C	*p*-value
Pre-operative	Full device	36.68 (0.39)	***p*** **< 0.001**
	Control	36.41 (0.34)	
Intra-operative (first 60 min)	Full device	36.33 (0.41)	***p*** **< 0.001**
	Control	35.9 (0.48)	
Intra-operative (overall)	Full device	36.63 (0.45)	***p*** **< 0.001**
	Control	36.21 (0.63)	
Post-operative	Full device	36.81 (0.37)	***p*** **< 0.001**
	Control	36.45 (0.57)	
Pre-operative	Popliteal	36.5 (0.31)	*p* = 1.00
	Sole	36.47 (0.48)	
Intra-operative (first 60 min)	Popliteal	36.49 (0.36)	*p* = 0.11
	Sole	36.33 (0.50)	
Intra-operative (overall)	Popliteal	35.99 (0.51)	*p* = 1.00
	Sole	35.99 (0.63)	
Post-operative	Popliteal	36.64 (0.36)	*p* = 0.09
	Sole	36.39 (0.48)	
Pre-operative	Full device	36.68 (0.39)	**vs. control** ***p*** **< 0.001** **vs. popliteal** ***p*** **= 0.04** **vs. sole** ***p*** **= 0.04**
	Control	36.41 (0.34)	**vs. popliteal** ***p*** **= 0.03** vs. sole *p* = 0.79
	Popliteal	36.5 (0.31)	vs. sole *p* = 1.00
	Sole	36.47 (0.48)	
Intra-operative (first 60 min)	Full device	36.33 (0.41)	**vs. control** ***p*** **< 0.001** **vs. popliteal** ***p*** **< 0.001** **vs. sole** ***p*** **< 0.001**
	Control	35.9 (0.48)	vs. popliteal *p* = 1.00 vs. sole *p* = 1.00
	Popliteal	36.49 (0.36)	vs. sole *p* = 0.11
	Sole	36.33 (0.50)	
Intra-operative (overall)	Full device	36.63 (0.45)	**vs. control** ***p*** **< 0.001** **vs. popliteal** ***p*** **= 0.003** **vs. sole** ***p*** **= 0.002**
	Control	36.21 (0.63)	**vs. popliteal** ***p*** **= 0.02** vs. sole *p* = 0.51
	Popliteal	36.41 (0.51)	vs. sole *p* = 1.00
	Sole	36.33 (0.63)	
Post-operative	Full device	36.81 (0.37)	**vs. control** ***p*** **< 0.001** **vs. popliteal** ***p*** **= 0.01** **vs. sole** ***p*** **< 0.001**
	Control	36.45 (0.57)	vs. popliteal *p* = 0.69 vs. sole *p* = 1.00
	Popliteal	36.64 (0.36)	vs. sole *p* = 0.09
	Sole	36.39 (0.48)	

Table 2 - A two-sided paired t-test with post-hoc Bonferroni corrections was performed to compare mean perioperative temperatures between groups. Standard deviation is presented in parentheses. Significant differences are bolded. Abbreviations: *FAW* (forced air warming), popliteal (popliteal group), sole (sole group)

**Fig. 4:**
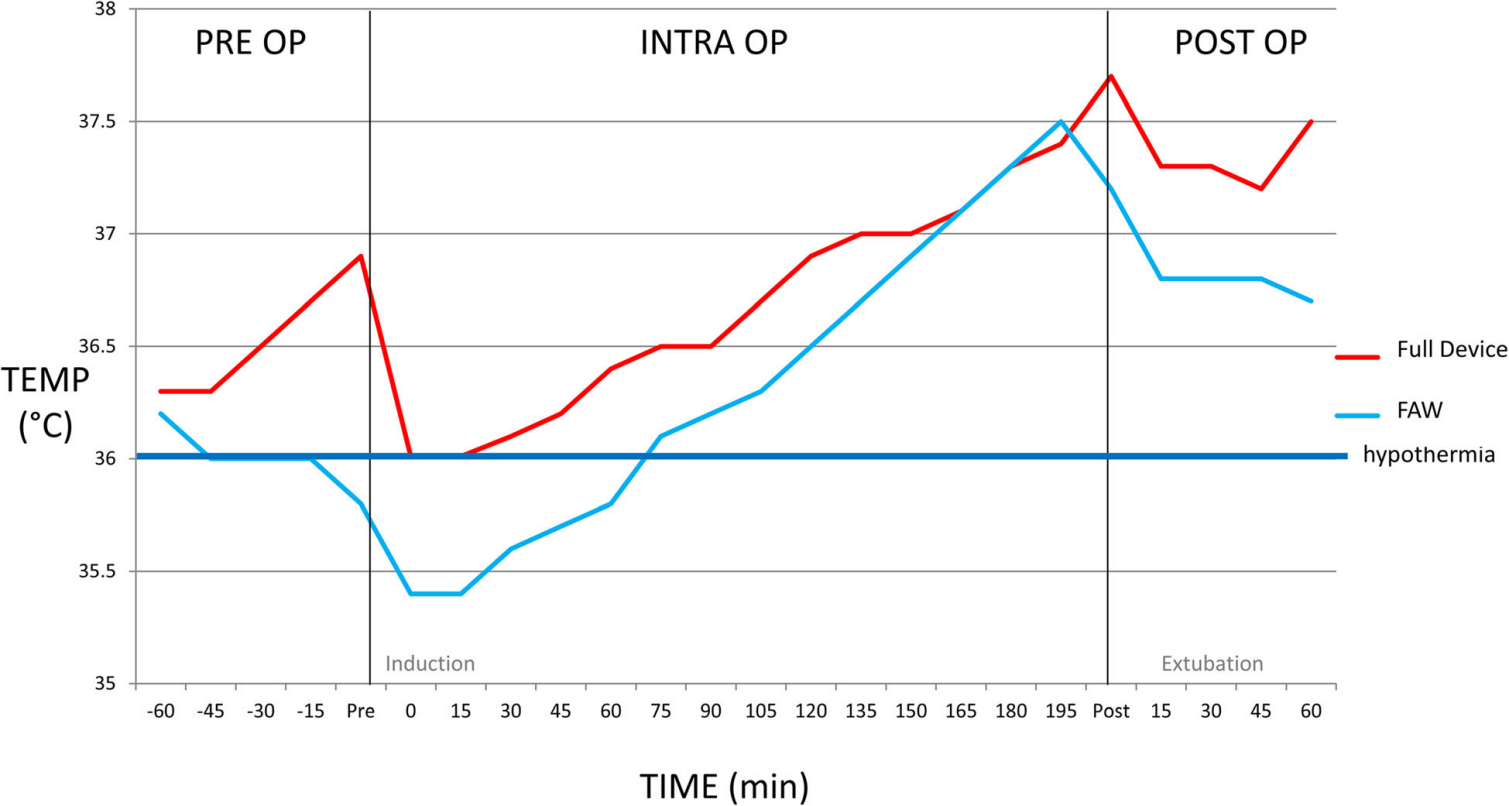
Comparison in the same patient. Figure 4 shows the temperatures (temp) recorded when the same patient underwent the same surgery on each breast at separate times. Once the patient was in the full device group and the other she was in the FAW group. Each phase is shown including pre op (preoperative), intra op (intraoperative) and post op (postoperative). The patient had higher temperatures in the full device group compared to when she was in the FAW group where she became hypothermic soon after induction

One patient had a unilateral mastectomy with the full device but then was required to have the contralateral side removed at a later date where she was randomly allocated to the FAW group. This allowed the comparison of the full device directly with the FAW within the same patient undergoing the same operation. Her perioperative temperatures are displayed in Fig. 4.

**Fig. 5:**
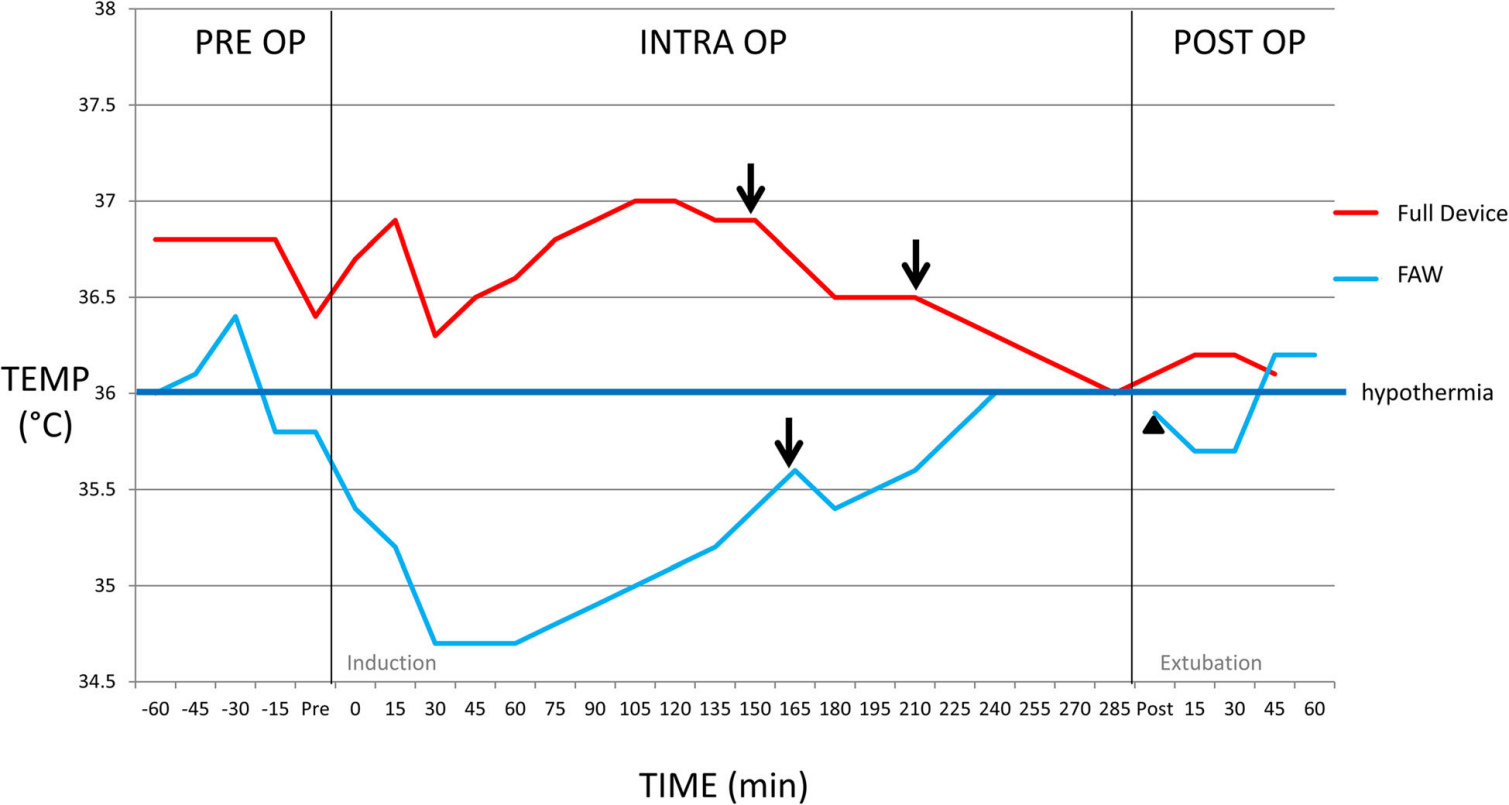
Case studies of surgery with patient turning. Figure 5 shows the temperatures (temp) recorded when two different patients underwent mastectomy with reconstruction using a pedicled latissimus dorsi muscle flap. Each phase is shown including pre op (preoperative), intra op (intraoperative) and post op (postoperative). The *down arrows* indicate times when the patient was completely undraped, turned over, re-prepped and draped. The *triangle* indications the FAW was transferred with the patient into and used through admission to the PACU. Each turn led to a reduction in mean temperature, however the patient with the full device maintained normothermia despite being turned over twice. The patient in the FAW group did not maintain normothermia

One patient in each group of stage I underwent reconstruction of the breast following mastectomy with a pedicled latissimus dorsi flap. The mean temperatures are displayed in Fig. 5. The full device was able to maintain normothermic temperatures throughout the perioperative period compared to the FAW patient that arrived in the post anesthesia care unit hypothermic (35.7 °C) and continued with FAW until discharge from the PACU.

### Thermal and general comfort scores

The preoperative thermal comfort scores were higher in the full device group (1.1 +/− 0.4) compared to the FAW group (−0.3 +/− 0.2), although the difference was small (*p* < 0.001). There was a mild benefit over the popliteal group (0.7 +/− 0.2) (*p* < 0.01) while the sole group had a wide range (−0.6 +/− 1.0) (*p* < 0.001). The postoperative thermal comfort scores showed a similar small benefit for the full device group (0.8 +/− 0.2) compared to the FAW (−0.1 +/− 0.2) (*p* < 0.001) and similar results in the popliteal (0.8 +/− 0.2) (*p* = 1.0) and sole groups (1.6 +/− 0.8) (*p* = 0.06). There were no or mild differences in preoperative general comfort scores between the full device (8.8 +/− 0.2), FAW (9.0 +/− 0.3) (*p* = 0.08), popliteal (8.4 +/− 0.2) (*p* < 0.01) or sole (8.0 +/− 0.6) (*p* = 0.01) groups. There were also no or mild differences in postoperative general comfort scores between the full device (8.0 +/− 0.3), FAW (8.0 +/− 0.5) (*p* = 1.0), popliteal (7.4 +/− 0.2) (*p* < 0.01) or sole (7.1 +/− 0.6) (*p* < 0.01) groups. Of interest was that patients who reported a cold preoperative thermal score were more likely to develop perioperative hypothermia (*p* = 0.03).

### Peri-operative complications

The mean estimated blood loss was significantly less in the full device group (106.9 +/− 20.2) compared to the FAW group (130.3 ml +/− 20.8) (*p* = 0.002). There were four wound infections in the FAW group including one abscess requiring drainage, one cellulitis requiring admission and intravenous antibiotics and two superficial wound infections requiring oral antibiotics. The full device group had a single infective complication, a case of superficial wound infection requiring oral antibiotics. There were two cases of seroma in the FAW group, none in the full device group. Overall, patients in the FAW group had higher rates of hypothermia associated wound complications (27.8%) than those with the full device (5.6%) although, given the small sample size, the difference was not statistically significant (*p* = 0.07). When the patients were analyzed according to whether or not they became hypothermic in the PACU, regardless of whether they were in the full device, the FAW, the popliteal group or the sole group they were significantly more likely (57.1%, 4 of 7) (*p* = 0.001) to develop postoperative wound complications compared to normothermic patients (8.5%, 4 of 47). That is, if a patient became hypothermic in the PACU, they were more likely to develop a wound complication, independent of the group they were in. The risk of developing a wound complication was more than 6 times higher if the patient was hypothermic in the postoperative period (RR = 6.71, 95% Confidence Intervals 0.11–0.86, *p* < 0.001). There was one tissue expander associated wound dehiscence in the device group. There were no cases of thermal injury.

#### Skin temperature

In all cases of device usage the skin temperature recorded at 15 min intervals intraoperative and postoperatively was 43 °C. In all cases of the device usage, preoperatively skin temperature reached the target of 43 °C within 30 min.

### The importance of heating both in the popliteal fossa and the sole of the feet

**Fig. 6:**
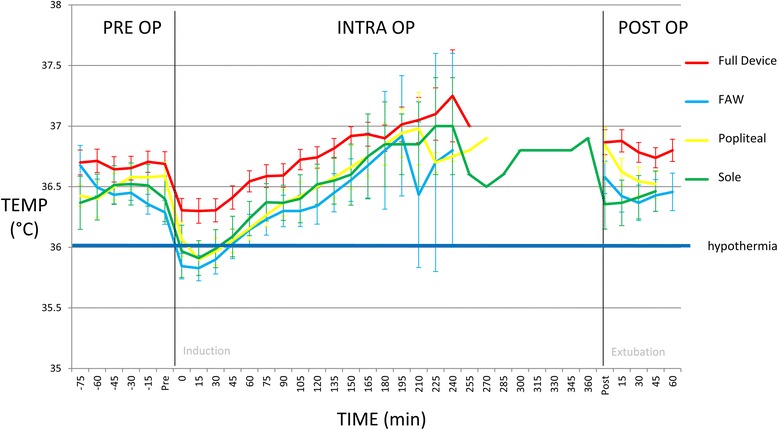
Perioperative temperatures in single heating treatment groups. Figure 6 shows the importance of heating in both areas for efficacy. The mean patient temperatures (temp) with standard error are shown for the full device (device, *n* = 18), FAW (*n* = 18), popliteal (pop fossa, *n* = 9) and sole (sole of foot, *n* = 9) groups. Each phase is shown including pre op (preoperative), intra op (intraoperative) and post op (postoperative). Both individual heating treatment groups had higher mean preoperative, early intraoperative and overall intraoperative temperatures compared to FAW although as a group the mean was still hypothermic after induction

The mean temperatures of all groups are displayed in Fig. 6. Patients in the full device group had significantly higher intra-operative temperatures within the first 60 min and lower incidence of perioperative hypothermia then both the popliteal and sole groups. Heating in only the popliteal fossa or sole of the feet regions led to significantly higher mean temperatures in the pre-operative period compared to FAW, but not significantly different intraoperative or post-operative temperatures. The incidence of perioperative hypothermia in the popliteal group (44%, *p* = 0.16) and sole group (55.6%, *p* = 0.39) was not significantly different to FAW (72%). There was one (of nine) patient in the popliteal and two (of nine) patients in the sole groups that had their intraoperative temperature rescued using FAW.

### The importance of the leg compression in the device

**Fig. 7:**
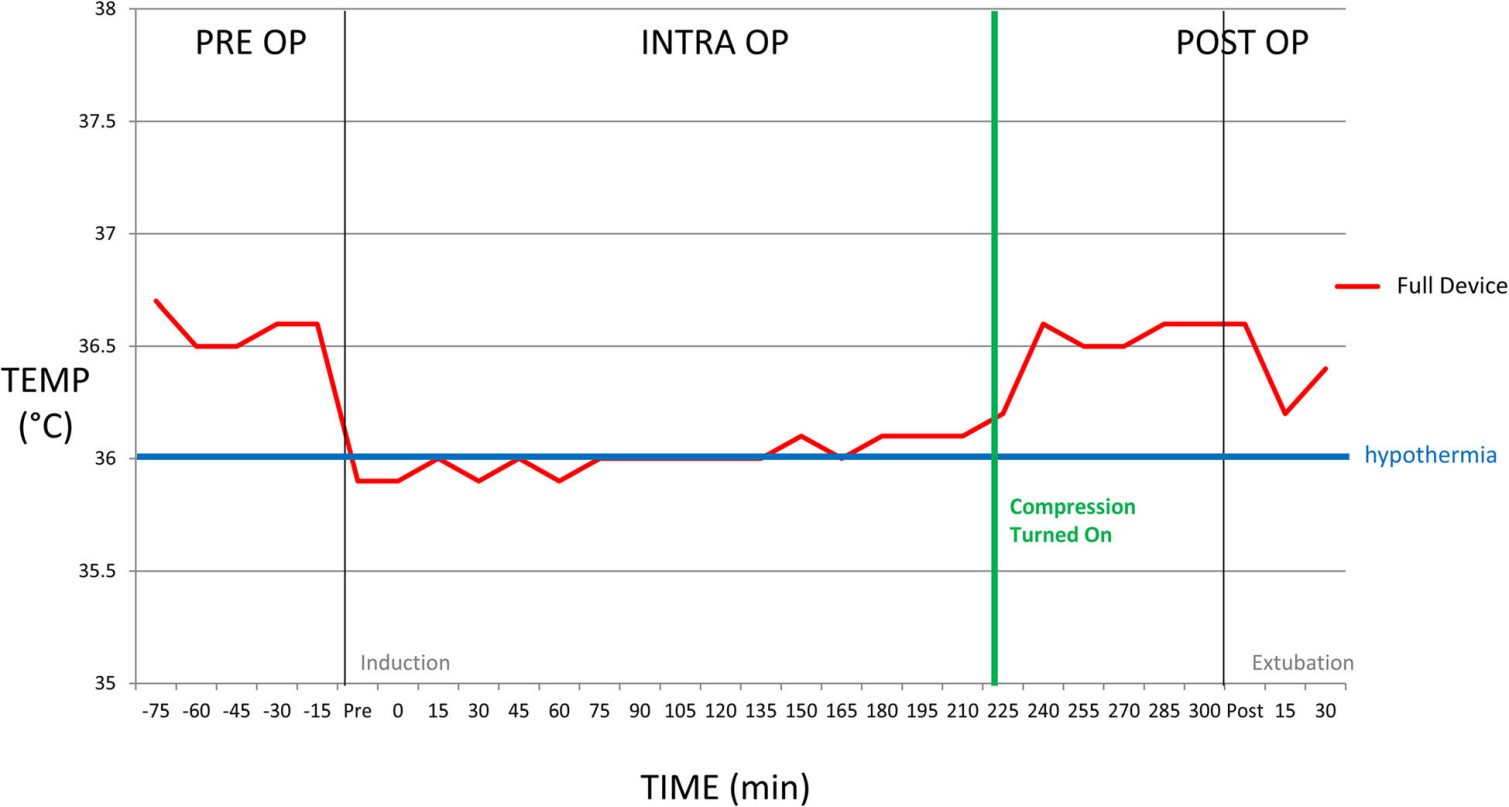
Compression is needed for device efficacy. Figure 7 shows the importance of the lower leg compression for full device efficacy. Each phase is shown including pre op (preoperative), intra op (intraoperative) and post op (postoperative). The temperatures (temp) recorded were hypothermic on induction and failed to rise until it was realized the lower leg compression had not been turned on. After turning on the patient’s temperature began to rise

We had one case, excluded from the cohort, which was found to not have the sequential calf compression turned on during the time out prior to surgery. This was realized intraoperatively and corrected. This then led to an increase in core temperature which is displayed in Fig. 7.

## Discussion

### A novel thermal compression device has higher mean perioperative mean temperatures and lower incidence of perioperative hypothermia compared to the current standard of care

Our study is a proof of concept of the potential usefulness of this device with statistically significant improvements in mean temperature and incidence of perioperative hypothermia, in each stage of surgery. Importantly, the full device showed benefit in the early intraoperative period, during which redistribution hypothermia occurs. Our research suggests that both areas of heating are needed for efficacy. Warming the peripheries preventatively is not necessarily a novel idea [10, 24]. It is the combination of the heating locations with lower leg compression that is unique. Further studies with larger cohorts, in other patient groups with large surgical exposure, will be useful to further investigate the clinical significance of improved perioperative temperatures with this device. Our study also suggests, in one patient, that the compression is essential for efficacy. Having a treatment group using the full device without compression would have been conducting practice against international guidelines for preventing peri-operative deep venous thrombosis [25, 26].

Reconstruction of the breast following mastectomy with a pedicled latissimus dorsi flap involves standing uncovered before surgery for reconstructive mark up, multiple turns during surgery between supine and prone as well as large parts of the body exposed. FAW is even more difficult under these circumstances. The full device was able to maintain normothermic temperatures in this setting despite twice undergoing complete undraping and repreparation of the surgical site. The FAW by comparison only had undraping and repreparation performed once but was hypothermic on arrival and at discharge from the PACU.

### Factors leading to inadvertent perioperative hypothermia

There are a number of opportunities for heat loss. Patients are poorly insulated in cold waiting areas. In the operating room they are naked, exposed and prepared with cold sterilization liquids. At anesthetic induction, impairment of the normal autonomic thermoregulatory controls causes vasodilatation and reduction in muscle tone leading to redistribution hypothermia as cold peripheral blood is circulated to the warm core within the first 30 min of induction [10, 27]. Our study also found that a patient reporting a preoperative cold thermal score was associated with IPH. A patient’s feeling of cold preoperatively may indicate that additional warming measures should be taken prior to being declared ready for transfer.

### Higher core temperatures in the perioperative period lead to better clinical outcomes

There is a lack of research to explain when the most critical period is for maintaining normothermia, but research supports that if IPH occurs it leads to poor outcomes [1, 5, 6]. Research also supports that even if normothermic, core temperatures further below 37 °C lead to poorer outcomes [28]. IPH should be avoided at all times and temperatures closest to 37 °C should be the aim for all phases of surgery. In the United States, compliance with the standards set by the Joint Commission and the Centers for Medicare and Medicaid Services is obtained if the patient is normothermic at the end of anesthesia (when the patient leaves the PACU) or if the patient received FAW. Compliance is therefore still possible with IPH or with FAW implemented incorrectly [29]. This leads to high compliance with standards but no improvement in clinical outcomes. The challenge for these governing bodies is to create standards for perioperative temperature measurement that, when complied with, lead to better clinical outcomes.

The device group had a trend towards a lower incidence of post-operative wound infections compared to the FAW group, although we recognize the study was not powered to detect this difference and a larger cohort is needed to determine its true effect. Incidence of hypothermia occurring in the PACU has already been shown to be a risk factor for wound infection with a relative risk of 6.0 [28]. This is comparable to the relative risk (6.0) of not giving perioperative antibiotics within 2 h before the surgery start time [28]. Our study showed that hypothermia in the postoperative period increased the risk of developing associated postoperative wound complications (infection or seroma) by 6.7 times compared to those without postoperative hypothermia. This may be partially explained by the direct correlation between core temperature and production of reactive oxygen intermediates by polymorphonuclear leucocytes [30]. Patients who do not receive active warming also have reduced lymphocyte activation and reduced IL1b and IL2 levels, compared to those that do [31]. There were less, but not significant, estimated blood losses in the device group compared to the FAW group in our study, and there was a higher incidence of post-operative seroma in the FAW group. In a meta-analysis, incidence of IPH increases the relative risk of transfusion by 22% [32]. Activated partial thromboplastin time and prothrombin time are prolonged in patients with lower temperatures, even if the patient is normothermic. There is an improvement in clotting that occurs as temperature increases up to 37 °C, with no significant additional benefit above 37 °C. Laboratory parameters are measured at 37 °C meaning abnormalities in clotting will not be detected when measured in hypothermic patients [33]. Active warming has also been tied to reduced cardiac morbidity and reduced length of stay in hospital [5, 34]. There were no incidences of cardiac morbidity in any of our cohorts. A larger powered cohort would be useful to further investigate whether the device’s higher mean temperatures and lower incidence of hypothermia lead to a reduction in other hypothermia associated complications.

### The novel thermal compression device avoids the compliance issues of the use of FAW.

A number of usability issues have been identified with FAW [7]. FAW obstructs the surgical site, especially in surgery of the core [7]. Because FAW relies on conduction and convection for heat transfer, a larger surface area is required for efficacy which is difficult in procedures involving the core. Current FAW require a conscious decision to implement. This requires the clinician to “opt in” to prevent perioperative hypothermia. A thermal compression device, tied to the implementation of sequential compression devices which are already placed routinely, will make it easier for compliance.

To date, all available alternatives to FAW rely on surface area coverage and therefore contain the same intrinsic disadvantages [35]. The only exception to this is a novel device using a vacuum in conjunction with heating of the palms of the hand of a single upper limb which failed to show benefit over the FAW [28, 36]. In addition, this device requires the inconvenience of immobilizing one upper limb and obstructs its intravenous access [28, 35]. In our study, the thermal compression device also demonstrated higher levels of thermal comfort and equivalent levels of general comfort. This can also be achieved with pre-operative FAW [12].

Since direct core heating is not possible, heating major blood vessels of the limbs, like the popliteal, can be effective [36]. These vessels remain close to the surface in patients with elevated body mass index. The blood flow through the popliteal fossa during rest is around 200 mL/min and increases to around 500 mL/min during calf compression, sufficient to alter cardiac output measurements [37, 38]. This device takes advantage of the increased blood flow in the area during compression to act as a peripheral warming pump into the core. Heat applied to the sole of the foot takes advantage of heat transfer via arteriovenous anastomoses [24]. Our study shows that the both heating areas with leg compression are needed.

#### Study limitations

The study was designed to test the feasibility and efficacy of the protoype device prior to further development. The numbers recruited were calculated to achieve power for the primary outcomes. Power was achieved for the reported outcomes, including the incidence of IPH, but was underpowered to detect the incidence of PACU hypothermia and the secondary outcomes of wound and other secondary surgical complications. We recognize larger cohort sizes would be needed to detect this difference. Our study was conducted in breast surgery only and should, in theory, be able to be extrapolated to other surgeries of the thorax and abdomen where the FAW is unable to be placed completely over those areas. It will be useful to test the device in other patient populations receiving surgery of different areas to understand the benefit of the device in those populations.

## Conclusions

In summary, a thermal compression device applied to the patient’s legs in the perioperative period improves patient temperature statistically significantly when compared to the current standard of care in this population group. This device has the potential for increased compliance of preventive measure to prevent hypothermia as implementation is uncomplicated and heating can start in the preoperative period or in the emergency room. Because it does not need a large surface area and does not get in the way for core surgical procedures it has the potential for improved clinical outcomes in these patients.

## Additional file

Additional file 1: Thermal compression device prototype technical description. (DOCX 13 kb)

## Abbreviations

ASA: American Society of Anesthesiologists
BMI: Body Mass Index
IPH: Inadvertent Perioperative Hypothermia
FAW: Forced Air Warming
PACU: Post Anesthesia Care Unit
