# Supplementary material for: A novel thermal compression device for perioperative warming: a randomized trial for feasibility and efficacy
Source: BMC Anesthesiol. 2017 Aug 11;17:102. doi: 10.1186/s12871-017-0395-2 (PMC5553896; doi:10.1186/s12871-017-0395-2)
Supplement: Additional file 1: — Thermal compression device prototype technical description. (DOCX 13 kb) [file 12871_2017_395_MOESM1_ESM.docx]

**Prototype Thermal Compression Device**

|  |  |
| --- | --- |

The prototype thermal compression device combines sequential compression of the calf and warming of the popliteal fossa and sole of the foot. For lower leg compression, a commercially available sequential compression device (SCD) (Kendall SCD, Covidien, Mansfield USA) was utilized with medium lower leg compression sleeves. This device was combined with four Homeothermic Blanket Systems (507220F, Harvard Apparatus, Holliston, Massachusetts, USA) for warming. The Homeothermic Blanket Systems (507220F, Harvard Apparatus, Holliston, Massachusetts, USA) each consisted of a control unit, a small (150mm x 200mm) warming blanket, and a thermal probe for temperature feedback. Separate systems were used for warming the popliteal fossa and sole of the foot locations.

The warming system is a closed loop control system where a temperature set point can be selected on the control unit and closed loop feedback and control occur between the temperature probe, control unit, and warming blanket. This allows the system to adjust the warming blanket temperature to ensure the delivery of appropriate thermal energy to the site for maximum warming while preventing any undesired complications due to the delivery of excess heat. Each warming site utilized a separate system with individual closed loop heating control to account for differences in warming blanket positioning, heat transfer, and other differences between the sites. In addition to the Homeothermic Blanket System (507220F, Harvard Apparatus, Holliston, Massachusetts, USA), a custom made sleeve was utilized to hold and position the warming blanket and thermal probe in the appropriate location. The sleeve positioned the thermal probe in contact with the warming location to take temperature readings of the skin for use in closed loop feedback control of the warming blanket. Each custom sleeve contained an insert to hold the thermal feedback probe directly against the skin. Each control unit was limited at 43°C.

The sleeves were positioned around the SCD for warming of the popliteal fossa and the sole of the foot and did not interfere with the normal functioning of the SCD.
